# Comparison of Chemical Composition between Kuromoji (*Lindera umbellata*) Essential Oil and Hydrosol and Determination of the Deodorizing Effect

**DOI:** 10.3390/molecules25184195

**Published:** 2020-09-13

**Authors:** Naoki Nanashima, Maiko Kitajima, Shizuka Takamagi, Miyuki Fujioka, Toshiko Tomisawa

**Affiliations:** 1 Department of Bioscience and Laboratory Medicine, Hirosaki University Graduate School of Health Sciences, 66-1 Hon-cho, Hirosaki, Aomori 036-8564, Japan; mfujioka@hirosaki-u.ac.jp; 2 Department of Nursing Sciences, Hirosaki University Graduate School of Health Sciences, 66-1 Hon-cho, Hirosaki, Aomori 036-8564, Japan; kitajima@hirosaki-u.ac.jp (M.K.); takamagi@hirosaki-u.ac.jp (S.T.); tmtott@hirosaki-u.ac.jp (T.T.)

**Keywords:** *Lindera umbellata*, kuromoji, deodorizing effect, essential oil, hydrosol, aromatherapy, linalool

## Abstract

Kuromoji (*Lindera umbellata*) is a tree that grows throughout Japan. The components of kuromoji essential oil have antitumor and aromatherapy effects. However, the composition of the hydrosol, obtained as a by-product of the essential oil process, is unknown. Furthermore, it is unknown whether kuromoji essential oil has a deodorizing effect. Therefore, the purpose of the current study was to compare the chemical composition of kuromoji essential oil and hydrosol, as well as evaluate the deodorizing effect of the former. The chemical composition of samples was evaluated using gas chromatography–mass spectrometry (GC-MS). Additionally, the deodorizing effect of Kuromoji essential oil was investigated with the detector tube method using ammonia, hydrogen sulfide, methyl mercaptan, and isovaleric acid. Linalool was the most abundant component in both the essential oil and hydrosol; however, its proportion was higher in the hydrosol (57.5%) than in the essential oil (42.8%). The hydrosol contained fewer chemical components, but higher proportions of trans-geraniol and ethanol. Moreover, the essential oil eliminated 50% of ammonia and 97.6% or more of isovaleric acid. Interestingly, linalool was soluble in the hydrosol and did not irritate the skin. This suggests that the hydrosol may be an effective foot care product.

## 1. Introduction

Kuromoji (*Lindera umbellate*) is a deciduous shrub that grows throughout Japan, and essential oils are made from its leaves and/or branches by steam distillation. It has been suggested that kuromoji essential oil has physiological effects and beneficial functions [1,2].

However, the components of the kuromoji hydrosol (aromatic distilled water), obtained as a by-product when refining the essential oil, are unknown. When distilling plants to extract essential oils used in aromatherapy, hydrosols are obtained from the distillation kiln [3,4]. Essential oils and hydrosols are similar in flavor/scent and properties because they are extracted from the same plant. They not only smell good, but also benefit the body and mind, and are therefore used for cosmetic and medicinal purposes. Even a single drop of essential oil is so potent that it cannot be applied directly to the skin or ingested as it can cause allergic reactions on the skin and mucous membranes [5]. Even when used for a steam bath or massage, essential oils must be diluted. Moreover, the use of some essential oils is contingent upon the condition of the user and the type of oil. However, hydrosols have few contraindications.

It is generally known that essential oils have a deodorizing effect, but little evidence exists regarding this function. Black cumin seed oil has a deodorizing effect against methyl mercaptan [6]. However, the deodorizing effect of kuromoji essential oil is unknown.

In this study, we compared the chemical components of kuromoji essential oil and kuromoji hydrosol. Furthermore, we evaluated the deodorizing effect of kuromoji essential oil against ammonia, hydrogen sulfide, and methyl mercaptan, which were used to emulate toilet and human waste odors; isovaleric acid was used as an index of body and foot odor. Although hydrosols do not have the immediate effect of drugs, they are gentle and effective. Therefore, we anticipate their application for deodorization purposes.

## 2. Results 

### 2.1. Comparison of the Chemical Composition of Kuromoji Essential Oil and Hydrosol

The results of the gas chromatography–mass spectrometry (GC-MS) analysis are presented in Table 1. Twenty-seven chemical compounds were detected in total. The essential oil and hydrosol contained 20 and 14 chemical compounds, respectively, of which seven compounds were detected in common. Many terpenoids were detected in both the kuromoji essential oil and hydrosol. The most common chemical compound detected was linalool, which constituted 42.8% and 57.5% of the essential oil and hydrosol, respectively. The second most common compound detected was 1,8-cineole, which accounted for 13.7% and 13.9% of the essential oil and hydrosol, respectively. β-myrcene and D-limonene were found to comprise 7.68% and 7.42%, respectively, of the essential oil. Furthermore, ethanol and trans-geraniol were only detected in the hydrosol, and they accounted for 18.1% and 3.71%, respectively. 

### 2.2. Deodorization Test

The results of the deodorization test are presented in Table 2. Kuromoji essential oil eliminated 50% and more than 97.6% of ammonia and isovaleric acid, respectively. However, kuromoji essential oil did not eliminate hydrogen sulfide and methyl mercaptan.

## 3. Discussion

In the present study, we compared the constituents of kuromoji essential oil and hydrosol. The results demonstrated that the most abundant constituent in both the essential oil and hydrosol was linalool (Table 1). Linalool is a terpenoid that is commonly present in plants, such as citrus [7]. It is known that linalool possesses several bioactivities, such as antibacterial [8], antiviral [9], antifungal [10], anti-inflammatory, and anticancer properties [11,12]. Additionally, it is a perfumery compound that is used in air fresheners [13].

Aromatherapy is the use of essential oils to promote the treatment of diseases and injuries. Linalool is frequently used in aromatherapy [14]. Kuromoji contains a large amount of linalool, and it is, therefore, expected to be used in aromatherapy. Although previous evidence has verified that the essential oil contains linalool, a higher percentage of linalool was detected in the hydrosol (Table 1). Therefore, the beneficial effects of the hydrosol were expected.

Maeda et al. (2012) reported that kuromoji essential oil contains linalool (65.78%), geranyl acetate (17.59%), geraniol (5.29%), and 1,8-cineole (2.34%) [11]. The content of geranyl acetate determined in the latter study was higher than that of the essential oil in the present study (1.02%), and was not detected in the hydrosol. In the present study, 1,8-cineole constituted 13.7% and 13.9% of the essential oil and hydrosol, respectively. These differences may be due to variations in the kuromoji production area and harvest season.

1,8-Cineole is abundantly present in eucalyptus plants and is an effective treatment for pediculosis [15], as well as an efficient biological herbicide [16], antimicrobial, and antioxidant [17]. Recently, several essential oil-derived components, such as 1,8-cineole, have been reported to inhibit influenza symptoms [18] and severity of coronavirus disease 2019 (COVID-19) [19,20,21]. Furthermore, some researchers are also investigating using 1,8-cineole as a new treatment for COVID-19 symptoms [22,23].

Plant terpenoids are widely used as deodorants due to their characteristic fragrance. Ammonia is responsible for a myriad of bad odors, including food waste, sweat, and urine, while isovaleric acid causes foot and age-related odors [24]. However, geraniol, found only in the hydrosol, is known as a fragrance compound [25]. Therefore, kuromoji hydrosol could be used to eliminate bad odors.

The results of the present study suggest that kuromoji oil may be effective in eliminating odors in general and particularly useful as a foot care product. The hydrosol and essential oil share similar main ingredients; therefore, the hydrosol could be used for spraying on masks, washing hands, or as an air freshener in the hospital and home environment.

## 4. Materials and Methods

### 4.1. Isolation of Kuromoji Essential Oil and Hydrosol

Fresh kuromoji leaves and branches were collected in June 2018 in the mountains near Hirakawa City, Japan. These samples were heated for 2 h to boiling point (100 °C). The hydro-distilled volatile component was subsequently collected and separated (upper layer: oil) from the aqueous portion (hydrosol). The kuromoji essential oil and hydrosol were purchased from Kojo Technology Co., Ltd. (Aomori, Japan).

### 4.2. Analysis of the Chemical Compounds Constituting Kuromoji Essential Oil and Hydrosol

The analysis of the constituents of kuromoji essential oil and hydrosol was carried out using GC-MS (QP2010 Plus; Shimadzu Corp., Kyoto, Japan). The GCMS-QP2010 Plus was equipped with a DB-5ms column (Agilent J&W; Agilent Technologies Japan Ltd., Tokyo, Japan) and a Stabilwax column (Shimadzu) for the analysis of the essential oil and hydrosol, respectively. The column oven temperature was programmed as follows: 40 °C for 2 min, increased by 10 °C/min afterward till the temperature reached 240 °C. The constituents were identified by matching computer-generated mass spectral data with the National Institute of Standards and Technology (NIST) mass spectral database. These experiments and analyses were performed at Kohga International Trading Co., Ltd. (Fukuoka, Japan).

### 4.3. Deodorization Test

One-liter Erlenmeyer flasks were charged with malodorous substances, such as ammonia (150 ppm), hydrogen sulfide (20 ppm), methyl mercaptan (5 ppm), and isovaleric acid (50 ppm). After adding 1 g of kuromoji essential oil, the mixture was left to stand at room temperature for 30 min. Thereafter, the residual concentration of each substance in the flask was measured using a kitagawa gas detector tube (Komyo rikagaku kogyo K.K., Kanagawa, Japan) connected to an aspirating pump (AP-20) and used to calculate the deodorizing rate. These experiments and analyses were performed once for each odor at Biostir Inc. (Osaka, Japan).

## 5. Conclusions

In this study, the components of kuromoji essential oil and hydrosol were analyzed. Linalool was the most common constituent of kuromoji essential oil (42.8%) and hydrosol (57.5%). The second most common constituent was 1,8-cineole, which was present in approximately equal proportions in both kuromoji oil and hydrosol.

Collectively, the chemical components had an odor-eliminating effect, with kuromoji oil eliminating more than 97.6% of isovaleric acid and 50% of ammonia.

The main components of kuromoji hydrosol are similar to that of the essential oil. However, the hydrosol is less irritating to the skin; therefore, it is expected to be used as an effective foot care product, as well as for other applications related to deodorization.

## Figures and Tables

**Table 1:** Chemical composition of kuromoji essential oil and hydrosol.

Compounds	Oil (%)	Hydrosol (%)
Ethanol	-	18.1
2,2,6-Trimethyl-6-vinyltetrahydro-2*H*-pyran	-	0.1
6,6-Dimethyl-2-methylenebicyclo [3.1.1]heptane	2.84	-
Camphene	2.33	-
β-Pinene	1.31	-
β-Myrcene	7.68	0.06
β-Ocimene	1.35	-
o-Cymene	2.01	-
d-Limonene	7.42	-
1,8-Cineole	13.7	13.9
Ocimene	1.65	-
β-cis-Ocimene	3.36	-
6-Methyl-5-hepten-2-ol	-	0.09
2-(5-Methyl-5-vinyltetrahydro-2-furanyl)-2-propanol	-	1.42
Linalool oxide	-	0.82
Fenchone	2.36	-
Linalool	42.8	57.5
DL-Camphor	1.07	-
(−)-4-Terpineol	1.33	1.29
α-Terpineol	1.46	1.53
Dihydrocarvone	3.88	0.44
d-Carvone	0.71	-
Borneol	-	0.68
(*R*)-(+)-β-Citronellol	-	0.33
trans-geraniol	0.97	3.71
Bornyl acetate	0.83	-
Geranyl acetate	1.02	-
Total	100	100

**Table 2:** Deodorizing effect of kuromoji essential oil.

Substance	Deodorizing Activity (%)
Ammonia	50
Hydrogen sulfide	0
Methyl mercaptan	0
Isovaleric acid	97.6<
